# The Error-Related Negativity (ERN) in Anxiety and Obsessive-Compulsive Disorder (OCD): A Call for Further Investigation of Task Parameters in the Flanker Task

**DOI:** 10.3389/fnhum.2021.779083

**Published:** 2021-12-09

**Authors:** Lilianne M. Gloe, Courtney C. Louis

**Affiliations:** Department of Psychology, Michigan State University, East Lansing, MI, United States

**Keywords:** ERN (error-related negativity), obsessive-compulsive disorder, anxiety, Flanker task, task parameters

## Introduction

The study of biomarkers and endophenotypes has proliferated in human clinical neuroscience in recent decades, with the hope that specific physiological signals may hold unique information for assessing psychopathology. Additionally, many researchers have sought to understand functional relationships between psychopathology and physiological phenomena to illuminate potential mechanisms and impacts of psychopathology. Both endophenotype and functional approaches have been used to interpret how anxiety^1^ and obsessive-compulsive disorder (OCD) relate to the error-related negativity (ERN), a negative-going signal that peaks within 100 ms after error commissions at frontocentral recording sites measured via electroencephalogram (EEG; Gehring et al., 2012; for reviews: Moser et al., 2013; Riesel, 2019). The functional significance of the ERN continues to be debated (for prominent theories, see Holroyd and Coles, 2002; Yeung et al., 2004; Holroyd et al., 2005; Gehring et al., 2012). However, it is generally accepted that the ERN is implicated in the error-monitoring process (Gehring et al., 2012). The ERN is likely generated by the dorsal region of the anterior cingulate cortex (ACC), an area highly responsive to conflict (Carter et al., 1998; Taylor et al., 2007), and influenced by activity in motor areas, such as the pre-supplementary motor area (pre-SMA; Hochman et al., 2009; Iannaccone et al., 2015). Meta-analytic evidence has demonstrated that the ERN is enhanced for those with high anxiety and OCD (for review: Moser et al., 2013, 2016; Riesel, 2019), and has highlighted the ERN as a promising physiological signal to understand these disorders.

Importantly, however, heterogeneity in the magnitude of the anxiety/OCD-ERN association has been identified (for review: Saunders and Inzlicht, 2020). Some of this variability in adults has been explained by moderators, such as sex and anxiety symptom dimensions (for review: Moser et al., 2016). Despite this, unexplained heterogeneity in effect sizes remains between studies. Further, a recent meta-analysis found that the magnitude of these effects is influenced by publication bias (Saunders and Inzlicht, 2020), suggesting that findings may be even more heterogeneous than indicated by the published literature. Therefore, additional moderators are likely present, influencing effect sizes and the utility of the ERN as an individual difference metric (Clayson et al., 2021).

One class of moderators that requires further elucidation are task parameters of the Flanker task, a commonly used task to elicit the ERN in the context of anxiety and OCD. The Flanker task is a speeded two-choice response task (Eriksen and Eriksen, 1974). In the traditional version of the task, participants are asked to respond to a center letter (i.e., the target) in the presence of several flanking distractor letters that can either be congruent (e.g., HHHHH) or incongruent (e.g., HHSHH) with the target. Notably, many studies have used a version of the Flanker task with modified arrow stimuli (e.g., <<<<< and <<><<). Although the Flanker task is a commonly used task in studies of the anxiety/OCD-ERN association, there is great heterogeneity in the task parameters used across studies. We consider how common variations in Flanker task parameters may influence the magnitude of the anxiety/OCD-ERN association.

## Task Manipulations and Anxiety/OCD-ERN Association

### The Role of Feedback in the Anxiety/OCD-ERN Association

One such manipulation of the Flanker task that varies between studies of anxiety/OCD and the ERN is the frequency and content of performance feedback provided to participants during the task. Many studies of anxiety/OCD and the ERN use a version of the task that provides indicators of performance at block breaks (e.g., Riesel et al., 2011; Klawohn et al., 2014; Weinberg et al., 2015). If participants fall below an accuracy threshold (e.g., <80% accuracy) they are told to respond more accurately following a block of trials. Alternatively, if performance falls above a particular accuracy threshold (e.g., >90%), participants are told to respond faster following a block of trials. Some versions of the task provide performance-based feedback after each trial (e.g., Cavanagh and Allen, 2008; Endrass et al., 2010; Xiao et al., 2011). Additionally, researchers have also used a paradigm in which no performance feedback is provided (e.g., Moran et al., 2012; Schroder et al., 2017; Riesel et al., 2019a,b).

In non-clinical samples, task instructions that emphasize accuracy over speed result in an enhanced ERN (Gehring et al., 1993; Arbel and Donchin, 2009, but see also Coleman et al., 2018). In contrast, when task instructions emphasize speed over accuracy, the ERN amplitude is diminished (Gehring et al., 1993). In studies of anxiety and OCD, performance-based feedback may moderate the anxiety/OCD-ERN association by altering relative emphasis on speed and accuracy. Riesel et al. (2019a,b) demonstrated that when accuracy was emphasized through task instruction and trial-to-trial feedback, healthy controls experienced an increase in the ERN, such that it was not significantly different from participants diagnosed with OCD. On the other hand, an enlarged ERN in OCD was still found in comparison to healthy controls when speed was emphasized through task instruction and trial-to-trial feedback (Riesel et al., 2019a,b). These findings provide compelling evidence that the task's relative emphasis on accuracy or speed may alter comparisons between those with and without OCD. Riesel and colleagues argue that individuals with OCD may experience difficulty adapting to different contexts and present with a more fixed response style. Additionally, Olvet and Hajcak (2009) identified that the ERN-anxiety association was not significant when trial-to-trial accuracy-based feedback was provided; however, the association did emerge when no trial-to-trial feedback was given. Olvet and Hajcak (2009) argue that feedback may reduce the load of error monitoring by providing anxious individuals with feedback on their performance. Therefore, existing evidence suggests that trial-to-trial feedback may influence the magnitude of the ERN in both OCD and anxiety, such that emphasizing accuracy results in a dampened association.

While no studies have examined the effect of block-to-block feedback on the anxiety/OCD-ERN association specifically, we hypothesize that providing different levels of accuracy emphasis to individuals results in a dampening of the association in tasks that use block-to-block feedback in comparison to those that use no feedback. For example, if Participant A makes more errors across blocks than Participant B, Participant A will receive more accuracy-based feedback. If Participant A also has relatively few blocks where high accuracy is achieved and, thus, receives little feedback about the speed of their performance, accuracy would be over-emphasized in feedback relative to speed. In contrast, Participant B who makes either a few or a moderate number of errors across blocks would either (1) receive more speed-related feedback because of high block accuracy or (2) minimal feedback about accuracy or speed because of average accuracy on block performance. Thus, the sample could be relatively heterogeneous for the relative proportions of speed and accuracy feedback, creating systematic, unaccounted for variability in the anxiety/OCD-ERN association.

### Other Task Factors to Consider in the Anxiety/OCD-ERN Association

In addition to performance-related feedback, future work should examine the role of task stimuli and mode of response. In a preliminary study, Lin et al. (2015) examined whether the association between worry and the ERN differed between tasks that used vertically and horizontally presented arrows, given that both presentations are regularly employed in studies of anxiety and the ERN. They found that worry was only associated with the ERN when arrow stimuli were presented horizontally (Lin et al., 2015). Lin et al. (2015) interprets these findings in the context of the Compensatory Error Monitoring Hypothesis (CEMH), which states that an enhanced ERN reflects compensatory recruitment of cognitive resources due to the taxing effect of worry (Moser et al., 2013). Specifically, Lin et al. (2015) theorize that worry utilizes verbal resources that are also being drawn upon during the processing of horizontal representations (i.e., reading). It may be that the association between worry/GAD and the ERN is more likely to emerge with horizontal stimuli because they are more cognitively demanding for worriers than vertical stimuli (Lin et al., 2015). Notably, no studies have examined whether effect sizes are influenced by the use of arrows as opposed to letters for stimuli, despite both versions of the stimuli being employed regularly in this literature. In addition, no studies have investigated whether the stimuli orientation (i.e., vertical vs. horizontal) influence the magnitude of the ERN in OCD or other types of anxiety besides worry.

Additionally, Flanker task versions differ in response mappings, such that some studies require participants to respond with index fingers on each hand, thumbs on each hand or the index and middle fingers on the same hand. Lin et al. (2015) also found that when using the left and right index finger to respond, worry was only related to the ERN on trials where errors were made with the right index finger during horizontal stimuli presentations, irrespective of participant handedness. In line with CEMH, the authors discuss that the worry-ERN association is larger with right-handed errors due to enhanced conflict driven by left-hemisphere verbal processing during horizontal stimulus presentation (Lin et al., 2015). That is, because verbal processing is left-lateralized and right-handed responses are controlled by the left-hemisphere, this increased conflict during right-handed errors results in an enhanced ERN magnitude. Indeed, others have suggested that response mapping factors, such as hand of error and even finger of error, influence the amplitude of the ERN itself (Hochman et al., 2014). Yet, these factors have yet to be considered in the empirical literature examining the anxiety/OCD-ERN association.

Finally, many versions of the letter flanker task include “switch blocks,” in which the stimulus-response mapping from the previous block is reversed. For example, the first block of a task may instruct participants to use their index finger to respond when an “M” is the target (i.e., center letter), while on the second block (i.e., the switch block) participants may be asked to use their middle finger to respond when an “M” is the target. In a non-anxious sample, one study found that the ERN is enhanced during switch blocks in comparison to non-switch blocks, speculated to be reflective of enhanced response conflict (Schroder et al., 2012). That is, switch blocks may function as a task-switching component of the flanker, during which the inhibition of previously learned stimulus-response mappings is required (Schroder et al., 2012). Because the ERN magnitude is larger on switch blocks, there could be variability in the association between the anxiety/OCD and the ERN on switch blocks, specifically, that has yet to be uncovered. Therefore, it will be important for studies to consider switch blocks as a within-subject moderator of the anxiety/OCD-ERN association.

## Conclusion

An impressive body of work has been generated over recent decades examining the association between anxiety/OCD and the ERN. We argue that task parameters, namely feedback, task stimuli, mode of response, response mappings and switch blocks, will be a useful avenue to explore to ultimately enhance clinical theories of the anxiety/OCD-ERN association. We expect that task feedback that emphasizes accuracy would reduce the association between anxiety/OCD and the ERN. Further, we expect that worry, specifically, will have a stronger association with the ERN when responses are made with one's right hand and when stimuli are presented horizontally. Given the lack of literature, it is difficult to predict if other anxiety dimensions (i.e., somatic anxiety) and OCD will function similarly to worry. Finally, we advocate for greater investigation of switch blocks given previous findings indicating that blocks with greater conflict result in a larger ERN in healthy controls. From an endophenotype/biomarker perspective, efforts to use the Flanker task as a clinical assessment tool should use Flanker task versions that maximize the difference between those with and without anxiety or OCD. Additionally, across endophenotype/biomarker and functional perspectives, considering task manipulations may provide further insights into the mechanisms underlying anxiety/OCD-ERN association. By developing this knowledge, the depth and potential applications of this work will continue to burgeon to the benefit of clinical populations.

## Author Contributions

LG and CL each wrote sections of the manuscript, contributed to manuscript revision, and approved the submitted version.

## Funding

LG was supported by the National Science Foundation Graduate Research Fellowship Program (NSF GRFP, United States, grant number: DGE-1848739) while working on this manuscript. CL was funded by the National Institute of Health Research Supplement to Promote Diversity in Health-Related Research and the Ruth L. Kirschstein National Research Service Award Individual Predoctoral Fellowship to Promote Diversity in Health-Related Research (NRSA F31 – Diversity, United States, grant number: F31MH125604) while working on this manuscript.

## Conflict of Interest

The authors declare that the research was conducted in the absence of any commercial or financial relationships that could be construed as a potential conflict of interest.

## Publisher's Note

All claims expressed in this article are solely those of the authors and do not necessarily represent those of their affiliated organizations, or those of the publisher, the editors and the reviewers. Any product that may be evaluated in this article, or claim that may be made by its manufacturer, is not guaranteed or endorsed by the publisher.

## Footnotes

^1^In the text, we use the term “worry” to refer to a specific cognitive dimension of anxiety, and the term “anxiety” to refer to both worry and somatic dimensions of anxiety (Heller et al., 1997; Nitschke et al., 2001).
